# Serological testing for SARS-CoV-2 antibodies of employees shows low transmission working in a cancer center

**DOI:** 10.1371/journal.pone.0266791

**Published:** 2022-04-12

**Authors:** Jeffrey A. Meyerhardt, Hong Yue, Radosław P. Nowak, Lauren Brais, Chao Ma, Samantha Johnson, Joanna Harrod, Shourya S. Roy Burman, Lynn M. Hendrickson, Stephanie Fischinger, Galit Alter, William Hahn, Bruce E. Johnson, Eric S. Fischer

**Affiliations:** 1 Department of Medical Oncology, Dana-Farber Cancer Institute, Boston, MA, United States of America; 2 Department of Cancer Biology, Dana-Farber Cancer Institute, Boston, MA, United States of America; 3 Department of Biological Chemistry and Molecular Pharmacology, Harvard Medical School, Boston, MA, United States of America; 4 Ragon Institute of Massachusetts General Hospital, Massachusetts Institute of Technology, and Harvard University, Cambridge, MA, United States of America; 5 Broad Institute of Harvard and MIT, Cambridge, MA, United States of America; U.S. Food and Drug Administration, UNITED STATES

## Abstract

**Background:**

The COVID-19 pandemic led to emergency measures to continue patient care and research at a comprehensive cancer center while protecting both employees and patients. Determining exposure and infection rates with SARS-CoV-2 were important to adjust workplace policies over time.

**Methods:**

Dana-Farber Cancer Institute (DFCI) has over 7,000 employees. Participation was voluntary. After consent, participants completed questionnaire of demographics, exposures and risk factors for COVID-19 illness at each time point (baseline, 3, 6, and 12 months) along with blood draws for SARS-CoV-2 antibody testing. Primary measure was determination of titers of SARS-CoV-2 spike protein IgG over time.

**Results:**

In total, 745 employees enrolled from May 2020 to February 2021 (mean [SD] age, 40[14] years; 572[80%] women). From May to July 2020, 47 of 519 employees (9.2%, 95% confidence interval [CI] 6.7–12.0%) tested positive for SARS-CoV-2 spike protein IgG antibodies. Three months later, 40 of 428 employees had positive antibodies (8.5%, 95% CI 6.0–11.0%) with 17 newly positive. At month 6, 78.5% of participants reported having received at least one dose of vaccine and the positivity rate for those vaccinated was 98% (95% CI, 95–100%). Spike protein IgG titers for those vaccinated were 7.9 times higher than participants not vaccinated (median IgG titer = 0.28 for positive antibody but not vaccinated versus 2.2 for vaccinated) but demonstrate evidence of waning over time.

**Conclusions:**

SARS-CoV-2 antibody positivity remained less than 10% at a single comprehensive cancer center prior to vaccination and there is evidence of waning IgG titers over time after vaccination.

## Introduction

In December 2019, a series of acute respiratory illness were reported in Wuhan, Hubei Province, China [1]. A novel coronavirus, initially named severe acute respiratory syndrome coronavirus 2 (SARS-CoV-2), was identified as the cause of this disease by the Chinese Center for Disease Control and Prevention (CDC) [2]. The disease, designated as coronavirus disease 2019 (COVID-19) by the World Health Organization (WHO), rapidly spread to other cities of China and around the globe so that on March 11, 2020, the WHO recognized COVID-19 as a worldwide pandemic [3]. Due to the highly infectious nature of COVID-19 and high rate of hospitalizations, countries around the world declared states of emergency and recommended or mandated stay-at-home orders. On March 23, 2020, Governor Charles Baker of the Commonwealth of Massachusetts ordered all non-essential businesses to cease in person operations. The Dana-Farber Cancer Institute rapidly operationalized remote work for the majority of non-direct care clinical employees but maintained direct patient care throughout the pandemic.

Identifying individuals infected by SARS-CoV-2 who are able to transmit the disease was a major public health challenge. Given the wide variability in presenting symptoms of patients infected with the virus, including asymptomatic carrier status, there was great interest in defining the immunity levels of a work force, particular those involved in health care. Serological assays were rapidly developed in the initial months of the pandemic, with variability in sensitivity and specificity. A collaborative group of investigators at the Ragon Institute of Massachusetts General Hospital, Massachusetts Institute of Technology and Harvard University, the Dana-Farber Cancer Institute, and the Broad Institute of Harvard and MIT developed a quantitative ELISA serological assay that uses recombinant SARS-CoV-2 spike or N proteins to measure IgM, IgG and IgA antibodies in sera. Although similar in concept to many reported assays, this assay was quantitative, highly sensitive and was easily adapted to high throughput assays [4,5].

With the development of a highly sensitive, quantitative assay, investigators from the Dana-Farber Cancer Institute mounted a volunteer employee antibody screening study for SARS-CoV-2, with repeated testing planned at baseline, 3 months, 6 months and 12 months. At each testing time point, participants self-reported demographic information, exposures, symptoms, PCR test results for COVID-19, comorbidities, and after January 2021, date of receipt of COVID-19 vaccination. This report presents the trends seen in COVID-19 antibody response and associations with participant characteristics.

## Methods

### Participants

This study was performed at the Dana-Farber Cancer Institute with approval by the Institutional Review Board. This voluntary study was made available to any Dana-Farber employee. Employee supervisors were not informed of employee participation. Eligibility for participation included age at least 18 years old; employment at the Dana-Farber Cancer Institute (main campus or satellite facilities), no COVID-related symptoms or known active infection at time of initial enrollment; able and willing to provide COVID-19 infection history and potential exposure information; willingness to provide preferred email address for study contact; did not receive any live viral vaccine within 4 weeks of enrollment or inactivated viral vaccine within 2 weeks; no known history of immune deficiency syndrome or undergoing therapy which can suppress immune system; no receipt of intravenous immunoglobulin within the month prior to initial enrollment; and ability to consent. A waiver of documentation of informed consent was granted by the Dana-Farber Cancer Institute Institutional Review Board, though participants were provided elements of consent through HIPAA compliant Research Electronic Data Capture REDCap application [6] and acknowledged receipt and understanding prior to proceeding with eligibility questions.

After enrollment, participants completed a questionnaire that collected COVID-19 and other infectious diseases personal history, social distancing and masking practices, work-related exposures (specifically patient-facing versus non patient-facing exposure), other medical conditions, and demographic information. After completion of the questions, participants scheduled a phlebotomy blood draw appointment that was performed at the main campus in Boston.

Participants agreed to have repeat questionnaires and blood draw measurements at baseline, 3 months, 6 months and 12 months. Initially, the study was open for accrual for six weeks from May 2020 to July 2020. At the 3 and 6-month timepoints, new enrollments to the study were allowed and thus some participants at the time of this report were only eligible for 2 or 3 blood draws. Six months after the study opened, the Food and Drug Administration (FDA) granted emergency use authorization to the first COVID-19 vaccine. Dana-Farber began vaccinating employees in mid-December 2020. As such, by the 6-month blood draw for participants who enrolled at initial study launch, many of the participants were vaccine eligible and the questionnaire added questions regarding COVID-19 vaccination status.

### SARS-CoV-2 antibody assays

The assays were carried out in a research laboratory at the Dana-Farber Cancer Institute. The full-length spike protein of SARS-CoV-2 (S protein prefusion stabilized with furin site removed, expressed in TunaCHO) was purchased from LakePharma (Cat. 46328). Serum samples were heat inactivated by incubation at 56°C for 1 hour before carrying out the spike protein ELISA according to previously established standard operating procedures [5]. In short, 384-well ELISA plates (ThermoFisher #464718) were coated with 50 μl/well of 500 ng/ml SARS-CoV-2 S protein (LakePharma Cat. 46328) in coating buffer (1 capsule of carbonate-bicarbonate buffer (Sigma #C3041100CAP) per 100 mL Milli-Q H2O) for 30 minutes at room temperature. Plates were washed 3 times with 100 μl/well of wash buffer (0.05% Tween-20, 400 mM NaCl, 50 mM Tris pH 8.0 in Milli-Q H2O) using a Tecan automated plate washer. Plates were blocked by adding 100 μl/well of blocking buffer (1% BSA, 140 mM NaCl, 50 mM Tris pH 8.0 in Milli-Q H2O) for 30 minutes at room temperature. Plates were then washed as described above. 50 μl of diluted samples (in dilution buffer; 1% BSA, 0.05% Tween-20, 140 mM NaCl, 50 mM Tris (pH 8.0) in Milli-Q H2O) was added to the wells and incubated for 30 minutes at 37°C. Plates were then washed 5 times as described above. 50 μl/well of diluted detection antibody solution (HRP-anti human IgG,IgA or IgM; Bethyl Laboratory #A80-104P, A80-100P, A80-102P) was added to the wells and incubated for 30 minutes at room temperature. Plates were then washed 5 times as described above. 40 μl/well of TMB peroxidase substrate (Thermo Fisher #34029) was then added to the wells and incubated at room temperature for 3 minutes (IgG)), 5 minutes (αIgA and αIgM). The reaction was stopped by adding 40 μl/well of stop solution (1 M H2SO4 in Milli-Q H2O) to each well. Optical density (OD) was read at 450 nm and 570 nm on a PHERAstar FSX plate reader. The final data used in the analysis OD_450-570_ was calculated by subtracting 570 nm background from 450 nm signal.

### Data processing

The binary classification label of the data for the IgG, IgA and IgM ELISA assay (0 –negative, 1 –positive) was obtained by comparing the response to thresholds optimized using a control set obtained from Mass General Brigham Biobank (named MGB set) consisting of 68 SARS-CoV-2 positive (CoV2+) and 100 pre-pandemic healthy controls (CoV2-). We previously established that maximum sensitivity and specificity was obtained with threshold set as the mean_CoV2-_ + 3 standard deviation_CoV2-_ for the IgG (98.6% sensitivity, 100% specificity), IgA (73.9% sensitivity, 99.0% specificity) isotypes and mean_CoV2-_ + 2 standard deviation_CoV2-_ for the IgM (69.4% sensitivity, 95.2% specificity) isotype (S1 Fig). The numerical values for the thresholds used were 0.121 for IgG, 0.182 for IgA and 0.270 for IgM, above which sample was classified as an isotype positive. In reviewing the titers of all three IgG, IgA and IgM isotypes in the MGB set and this study using the established ELISA method, it was determined that the IgG isotype to contain most information on the past exposure to SARS-CoV-2 due to its lasting response after full seroconversion and therefore IgG titer became the focus our analysis [7].

Once processed, the study data were collected and managed using REDCap electronic data capture tools hosted by MGB HealthCare Research Computing, Enterprise Research Infrastructure & Services (ERIS) group. REDCap is a secure, web-based application designed to support data capture for research studies.

### Statistical analysis

Overall seroprevalence was estimated using Bayes rule based on the sensitivity and specificity of the ELISA test at each timepoint (baseline, 3, 6 and 12 months). Specifically, the seroprevalence $\hat{p}=\frac{\hat{\pi }-\left(1-{ϒ}_{spec}\right)}{{ϒ}_{sen}-\left(1-{ϒ}_{spec}\right)}$, 95% confidence interval is: $\frac{\hat{\pi }-\left(1-{ϒ}_{spec}\right)}{{ϒ}_{sen}-\left(1-{ϒ}_{spec}\right)}\;\pm 1.96\cdot \frac{\sqrt{\hat{\pi }\left(1-\hat{\pi }\right)}}{\sqrt{n}\cdot \left({ϒ}_{sen}-\left(1-{ϒ}_{spec}\right)\right)}$. Where $\hat{\pi }$ is the positive rate and n is the number of people who are tested. Binomial exact test was used to estimate seroprevalence when all tested subjects had positive results.

To examine the associations between risk factors and presence of positive antibodies, we performed logistic regression analysis at baseline and month 3, respectively. To avoid the effect of vaccine, we only included participants who were not vaccinated at their time of blood draw.

## Results

Between May 2020 and February 2021, 745 eligible employees enrolled in a longitudinal study following SARS-CoV-2 antibody responses. In December 2020, the initial roll-out of COVID-19 vaccine was instituted at the Dana-Farber Cancer Institute, initially to direct patient-facing employees followed by other staff members participating in on site work followed by remote only employees. At the planned 6-months time point, 79% of participants who were not lost to follow-up or withdrew had received at least one dose of the vaccine.

**Fig 1:**
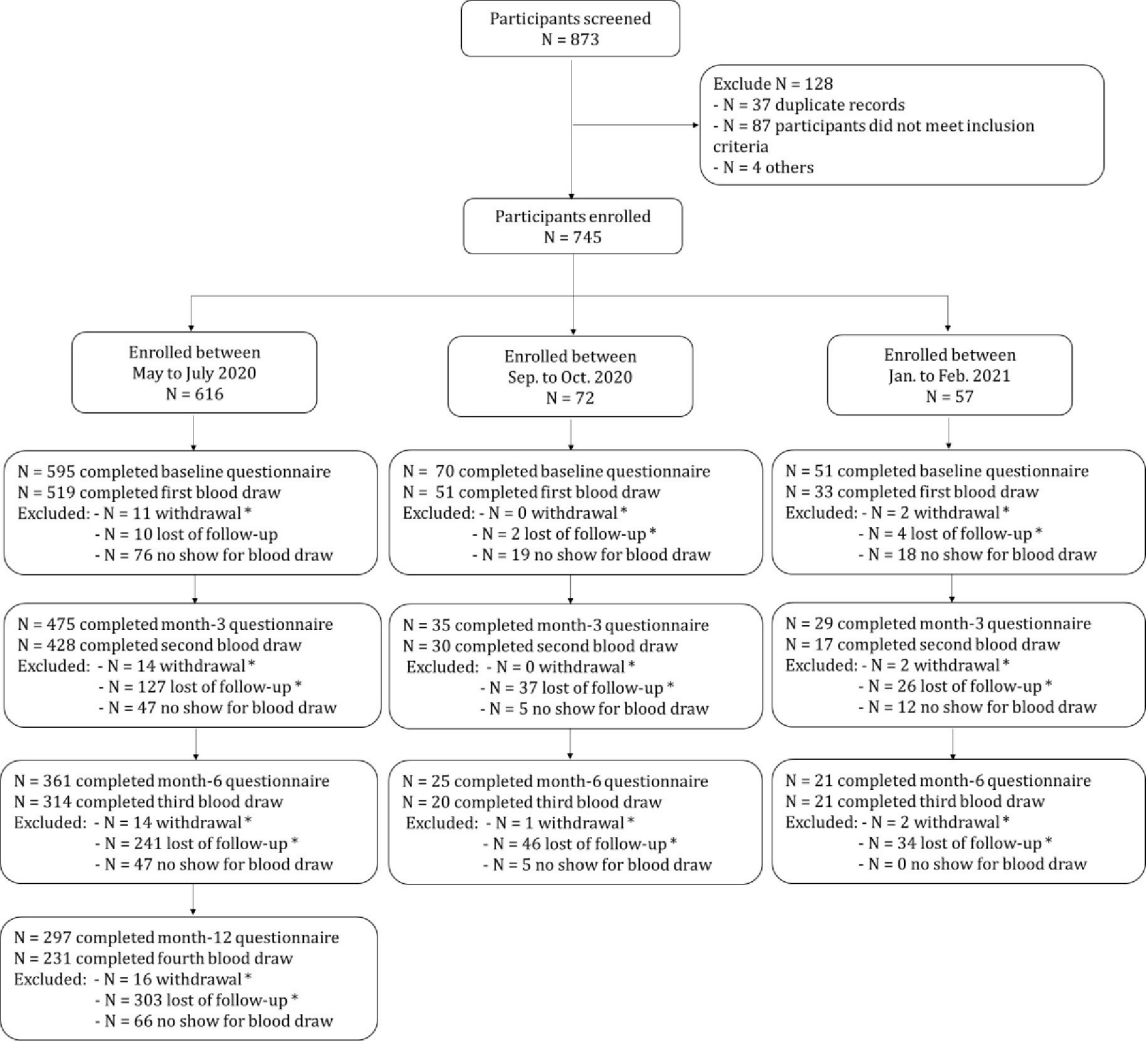
Consort diagram by the 3^rd^ open (Month 6 follow up). Legend: * Withdrawal and lost to follow-up are calculated from initial enrollment in that time period (ie. for those initially enrolled May to July 2020, 127 lost to followup at 3 month questionnaire was compared to N = 616). However, no show for blood draw is within the time point only (eg. for participants enrolled between May and July 2020, at the 3 month time point, 475 completed the questionnaire but only 428 had blood drawn, ie. 47 did not show for 3 month blood draw).

Fig 1 summarizes the flow of participants to date. New accrual of participants was allowed at the 3 and 6-months time periods, leading some participants to only be eligible to have had 2 or 3 follow-up questionnaires and blood draw. At each follow-up time point, there was notable number of participants who did not return the questionnaires and participate in the subsequent blood draws. As this was a voluntary employee study, we did not link registration to employee status per our institutional research guidelines. As a result, some lost to follow-up may no longer be Dana-Farber employees at subsequent time points or transitioned to fully remote work and thus not on site to easily participate in blood draw at our campus. Baseline participant characteristics are reported in Table 1. Updated percentages at each time point are provided both due to changing number of participants at each time point and potential changes to exposures and risk factors.

**Table 1:** Baseline participant characteristics.

	Enrolled from May to July 2020				Enrolled from Sep. to Oct. 2020			Enrolled from Jan. to Feb. 2021		
Factor	Baseline (N = 595)	Month-3 (N = 475)	Month-6 (N = 361)	Month-12 (N = 297)	Baseline (N = 70)	Month-3 (N = 35)	Month-6 (N = 25)	Baseline (N = 51)	Month-3 (N = 29)	Month-6 (N = 21)
Age, years										
• Median (IQR)	38 (29–52)	39 (29–53)	40 (30–54)	41 (30–55)	34 (25–51)	36 (26–51)	34 (26–51)	33 (26–46)	34 (26–47)	32 (27–46)
• Missing	7	6	5	5	1	1	0	1	2	1
Sex -N (%)										
• Female	471(79.3)	374(78.9)	286(79.4)	234(79.1)	58(84.1)	28(82.4)	21(84.0)	43(86.0)	22(81.5)	17(81.0)
• Missing	1	1	1	1	1	1	0	1	2	0
Race -N (%)										
• White	506(85.0)	408(85.9)	317(87.8)	259(87.2)	54(78.3)	27(79.4)	19(76.0)	41(80.4)	22(78.6)	17(81.0)
• Black	11(1.8)	10(2.1)	5(1.4)	4(1.3)	1(1.4)	0(0.0)	0(0.0)	1(2.0)	1(3.6)	0(0.0)
• Asian	50(8.4)	37(7.8)	23(6.4)	23(7.7)	10(14.5)	5(14.7)	5(20.0)	7(13.7)	4(14.3)	3(14.3)
• Others	28(4.7)	20(4.2)	16(4.4)	11(3.7)	4(5.8)	2(5.9)	1(4.0)	2(3.9)	1(3.6)	1(4.8)
• Missing	0	0	0	0	1	1	0	0	1	0
Smoking status -N (%)										
• Current smoker	11(1.8)	10(2.1)	8(2.2)	5(1.7)	0(0.0)	0(0.0)	0(0.0)	1(2.0)	1(3.6)	0(0.0)
• Ex-smoker	81(13.6)	65(13.7)	46(12.7)	42(14.1)	7(10.1)	5(14.7)	3(12.0)	8(15.7)	6(21.4)	3(14.3)
• Never smoker	503(84.5)	400(84.2)	307(85.0)	250(84.2)	62(89.9)	29(85.3)	22(88.0)	42(82.4)	21(75.0)	18(85.7)
• Missing	0	0	0	0	1	1	0	0	1	0
Covid-19 tests -N (%)										
• Tested	102(17.1)	170(35.8)	266(73.7)	152(51.4)	39(56.5)	22(64.7)	11(44.0)	46(90.2)	10(34.5)	5(23.8)
• Positive results (based on tested)	9(8.8)	0(0.0)	10(3.8)	1(0.66)	3(7.7)	0(0.0)	0(0.0)	8(17.4)	0(0.0)	0(0.0)
• Hospitalized due to Covid-19 (Based on positive results)	1(11.1)	----	0(0.0)	0(0.0)	1(33.3)	----	----	0(0.0)	----	----
• Missing	0	0	0	1	1	1	0	0	0	0
Travel history—N (%)										
• Travel outside of US since Nov 2019 (baseline questionnaire) or past 3 months (follow-up questionnaires)	166(27.9)	2(0.42)	2(0.55)	16(5.4)	22(31.9)	0(0.0)	0(0.0)	9(17.6)	0(0.0)	0(0.0)
• Missing	1	0	0	0	1	1	0	0	0	0
• Travel outside of MA since Jan 2020 (baseline questionnaire) or past 3 months (follow-up questionnaires)	405(68.1)	322(67.8)	155(42.9)	238(80.1)	52(75.4)	15(44.1)	16(64.0)	33(64.7)	14(48.3)	13(61.9)
• Missing	0	0	0	0	1	1	0	0	0	0
Area exposed when on campus—N (%)										
• Clinic or infusion area	216(36.3)	207(43.6)	165(45.7)	140(47.1)	28(40.0)	14(40.0)	13(52.0)	24(47.1)	15(51.7)	10(47.6)
• Inpatient floors	52(8.7)	50(10.5)	46(12.7)	41(13.8)	5(7.1)	4(11.4)	2(8.0)	4(7.8)	1(3.4)	1(4.8)
• Inpatient Special Pathogens Unit	19(3.2)	3(0.63)	10(2.8)	3(1.0)	0(0.0)	0(0.0)	0(0.0)	0(0.0)	0(0.0)	0(0.0)
• Office space	257(43.2)	247(52.0)	197(54.6)	179(60.3)	36(51.4)	23(65.7)	18(72.0)	27(52.9)	14(48.3)	11(52.4)
• Laboratory space	122(20.5)	121(25.5)	104(28.8)	89(30.0)	20(28.6)	14(40.0)	12(48.0)	7(13.7)	5(17.2)	3(14.3)
• Cafeteria	206(34.6)	208(43.8)	171(47.4)	156(52.5)	19(27.1)	8(22.9)	7(28.0)	19(37.3)	13(44.8)	9(42.9)
• Facilities floor	28(4.7)	19(4.0)	19(5.3)	17(5.7)	4(5.7)	3(8.6)	1(4.0)	3(5.9)	1(3.4)	0(0.0)
• Parking areas	188(31.6)	159(33.5)	115(31.9)	109(36.7)	15(21.4)	11(31.4)	5(20.0)	16(31.4)	7(24.1)	6(28.6)
Direct patient care–N (%)										
• Yes	247(41.5)	193(40.6)	144(39.9)	116(39.1)	31(45.6)	13(39.4)	10(40.0)	24(47.1)	10(35.7)	8(38.1)
• Missing	0	0	0	0	2	2	0	0	1	0
Hours for sleeping (night before questionnaire) -N (%)										
• Less than 5 hours	14(2.4)	14(3.0)	10(2.8)	10(3.4)	2(2.9)	1(2.9)	1(4.0)	4(7.8)	2(7.1)	1(4.8)
• 5–6 hours	170(28.6)	134(28.3)	98(27.2)	89(30.1)	25(36.2)	12(35.3)	8(32.0)	14(27.5)	9(32.1)	6(28.6)
• 7–8 hours	382(64.3)	304(64.1)	233(64.7)	182(61.5)	40(58.0)	20(58.8)	16(64.0)	32(62.7)	17(60.7)	14(66.7)
• 9+ hours	28(4.5)	22(4.4)	19(5.0)	15(5.1)	2(2.9)	1(2.9)	0(0.0)	1(2.0)	0(0.0)	0(0.0)
• Missing	1	1	1	1	1	1	0	0	1	0
History of prior Influenza infection -N (%)	261(44.0)	211(44.6)	158(43.8)	128(43.1)	37(53.6)	19(55.9)	15(60.0)	22(43.1)	15(51.7)	9(42.9)
• Missing	2	2	0	0	1	1	0	0	0	0
Flu vaccine -N (%)	582(97.8)	469(98.7)	354(98.1)	293(98.7)	57(82.6)	29(85.3)	21(84.0)	50(98.0)	28(96.6)	21(100.0)
• Missing	0	0	0	0	1	1	0	0	0	0
Medical history -N (%)										
• Diabetes	13(2.2)	10(2.1)	6(1.7)	6(2.0)	4(5.7)	2(5.7)	2(8.0)	0(0.0)	0(0.0)	0(0.0)
• Hypertension	37(6.2)	31(6.5)	25(6.9)	21(7.1)	2(2.9)	1(2.9)	0(0.0)	2(3.9)	1(3.4)	1(4.8)
• Cardiovascular	9(1.5)	7(1.5)	7(1.9)	4(1.3)	0(0.0)	0(0.0)	0(0.0)	0(0.0)	0(0.0)	0(0.0)
• Seasonal Allergies	251(42.2)	201(42.3)	147(40.7)	119(40.1)	30(42.9)	14(40.0)	12(48.0)	14(27.5)	7(24.1)	4(19.0)
• Asthma	54(9.1)	37(7.8)	29(8.0)	21(7.1)	4(5.7)	2(5.7)	2(8.0)	3(5.9)	1(3.4)	1(4.8)
• Chronic lung disease	1(0.17)	0(0.0)	0(0.0)	0(0.0)	0(0.0)	0(0.0)	0(0.0)	0(0.0)	0(0.0)	0(0.0)
• Chronic kidney disease	1(0.17)	1(0.21)	1(0.28)	1(0.34)	0(0.0)	0(0.0)	0(0.0)	0(0.0)	0(0.0)	0(0.0)
• Liver disease	1(0.17)	1(0.21)	0(0.0)	1(0.34)	1(1.4)	1(2.9)	0(0.0)	0(0.0)	0(0.0)	0(0.0)
• Cancer	17(2.9)	13(2.7)	9(2.5)	5(1.7)	1(1.4)	1(2.9)	1(4.0)	3(5.9)	2(6.9)	2(9.5)
• Autoimmune disease	21(3.5)	18(3.8)	14(3.9)	12(4.0)	4(5.7)	2(5.7)	2(8.0)	2(3.9)	1(3.4)	1(4.8)
• Pregnant	3(0.50)	2(0.42)	2(0.55)	2(0.67)	1(1.4)	0(0.0)	0(0.0)	0(0.0)	0(0.0)	0(0.0)
• None of the above	278(46.7)	223(46.9)	173(47.9)	142(47.8)	31(44.3)	17(48.6)	11(44.0)	31(60.8)	17(58.6)	13(61.9)

N = sample size; IQR = Interquartile range; US = United States; MA = Massachusetts.

### SARS-CoV-2 antibody responses

**Fig 2:**
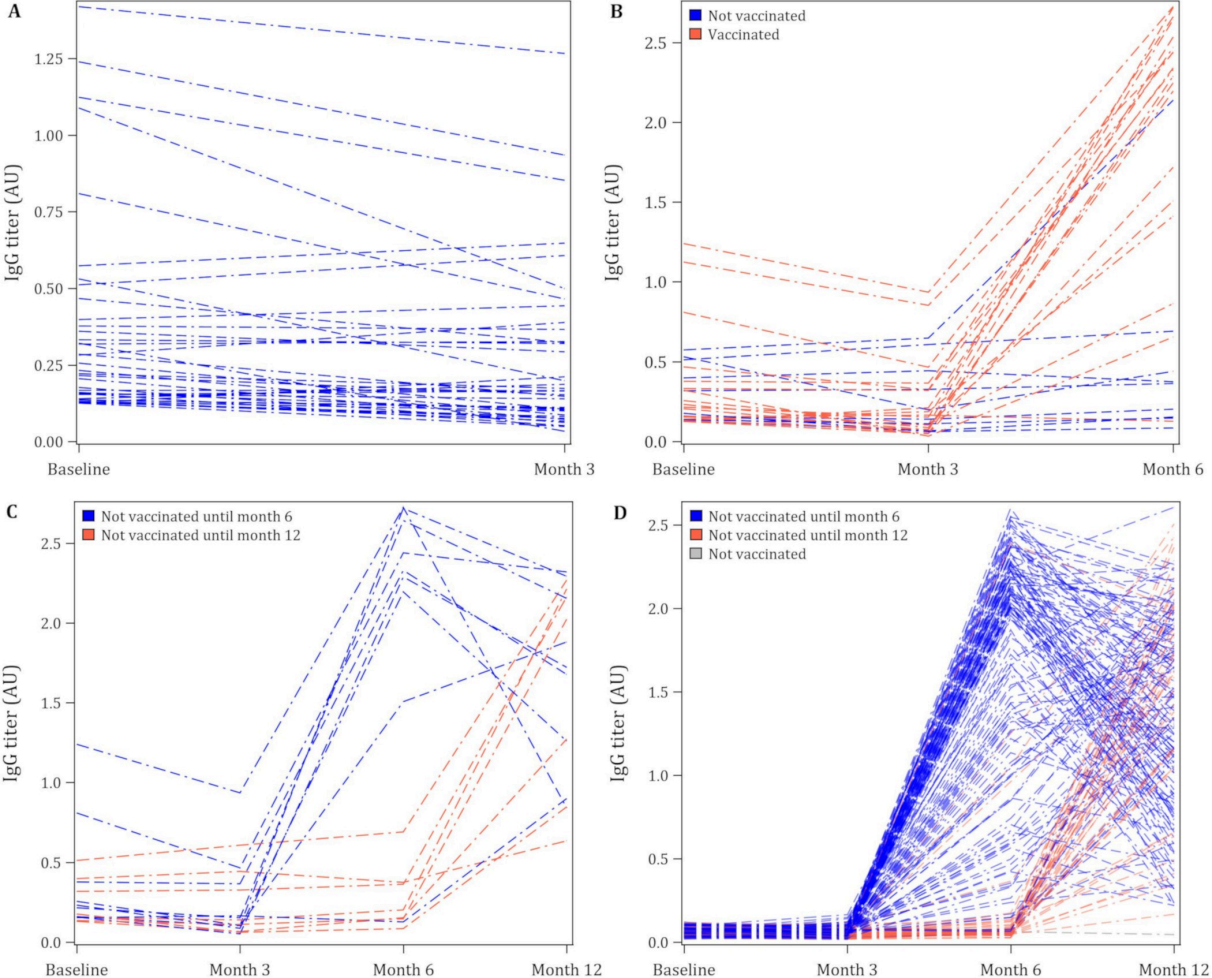
Spaghetti plots for participants positive IgG at baseline (A-C) and negative IgG at baseline (IgG) and subsequent follow-up levels. (A) Participants positive at baseline and had follow-up blood at month 3 (B) Participants positive at baseline and had follow-up bloods at months 3 and 6 (C) Participants positive at baseline and had follow-up bloods at months 3, 6 and 12 (D) Participants negative at baseline and had follow-up bloods at months 3, 6 and 12. Legend: AU = arbitrary units–the measurement is a subtraction of 570 nm background from 450 nm absorbance signal.

In the first 3 months of the study (May and July 2020) 47 of 519 participants (9.2%, 95% confidence interval [CI] 6.7–12.0%) were seropositive for spike protein IgG. Of those having blood testing 3 months after the start of the study, between September and October 2020, 40 of 479 participants (8.5%, 95% CI 6.0–11.0%) were seropositive. Of those testing positive in May or July of 2020 who had a repeat blood draw, 23 of 37 participants (63%, 95% CI 47–79%) remained positive at month 3 (Fig 2). Eleven of 387 seronegative from May to July 2020 with month 3 blood draw converted to seropositive (2.9%, 95% CI 1.2–4.6%).

During the 6-months blood draw performed from January to February 2021, 377 participants had blood draws (314 who enrolled between May and July 2021, 30 enrolled between September and October 2020, and 33 enrolled between January and February 2021). Of those participants, 81 (21%) reported that they had not been vaccinated. Since the questionnaire may have been completed up to 4 weeks prior to blood draw, it is possible a small percentage were vaccinated in the interim. However, of the 81 participants who reported not being vaccinated, 31% (95% CI 21–41%) were IgG seropositive. A total of 210 participants had blood draws at all 4 time points. Only one (0.5%) reported not being vaccinated by 12 months; the seropositivity rate for that participants at 12 months was 0%.

Once vaccinations were available to employees, the seropositivity rate dramatically increased. Of those having blood drawn from January to February 2021, 296 of 377 (79%) reported receiving at least one dose of COVID vaccine. For the 236 having received 2 doses, the seropositivity rate was 100% (95% CI, 99–100%), both for those who received 2^nd^ dose within 14 days of blood draw (103 participants) and those with 2^nd^ dose at least 14 days from blood draw (133 participants). For those vaccinated with only one dose (at the time of the blood draw, the Pfizer and Moderna vaccines were the only vaccines available and both required 2 doses), 32 of 32 (100%, 95% CI 89–100%) were seropositive if vaccinated at least 14 days from blood draw and 19 of 28 (69%, 95% CI 51–86%) were seropositive if vaccination was less than 14 days from blood draw.

At 12 months, all but 3 participants from baseline enrollment who completed 12 month questionnaire and participated in the blood draw (231 participants) were unvaccinated. Of those vaccinated, 100% (95% CI 99–100%) were seropositive. Of the 3 participants not vaccinated at the 12 month blood draw, 2 (68%, 95% CI 14–100%) were seropositive.

**Fig 3:**
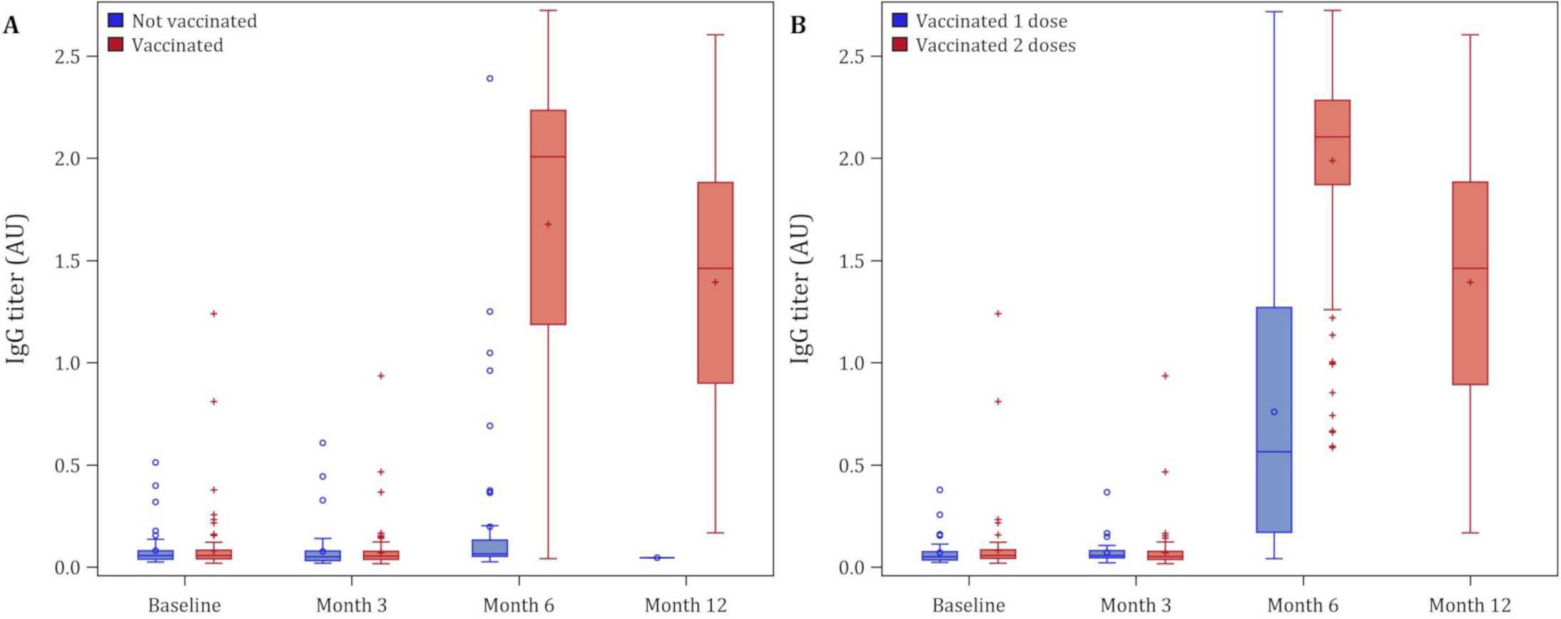
Box plot for participants who got blood tested at baseline, months 3, 6 and 12. (A) All participants; (B) Vaccinated with at least one dose. Legend: The bottom and top edges of the box indicate the IQR, the marker inside the box indicates the mean value, the line inside the box indicates the median value. The upper and lower fences are maximum and minimum values, respectively, points beyond upper and lower fences are outliers.

**Fig 4:**
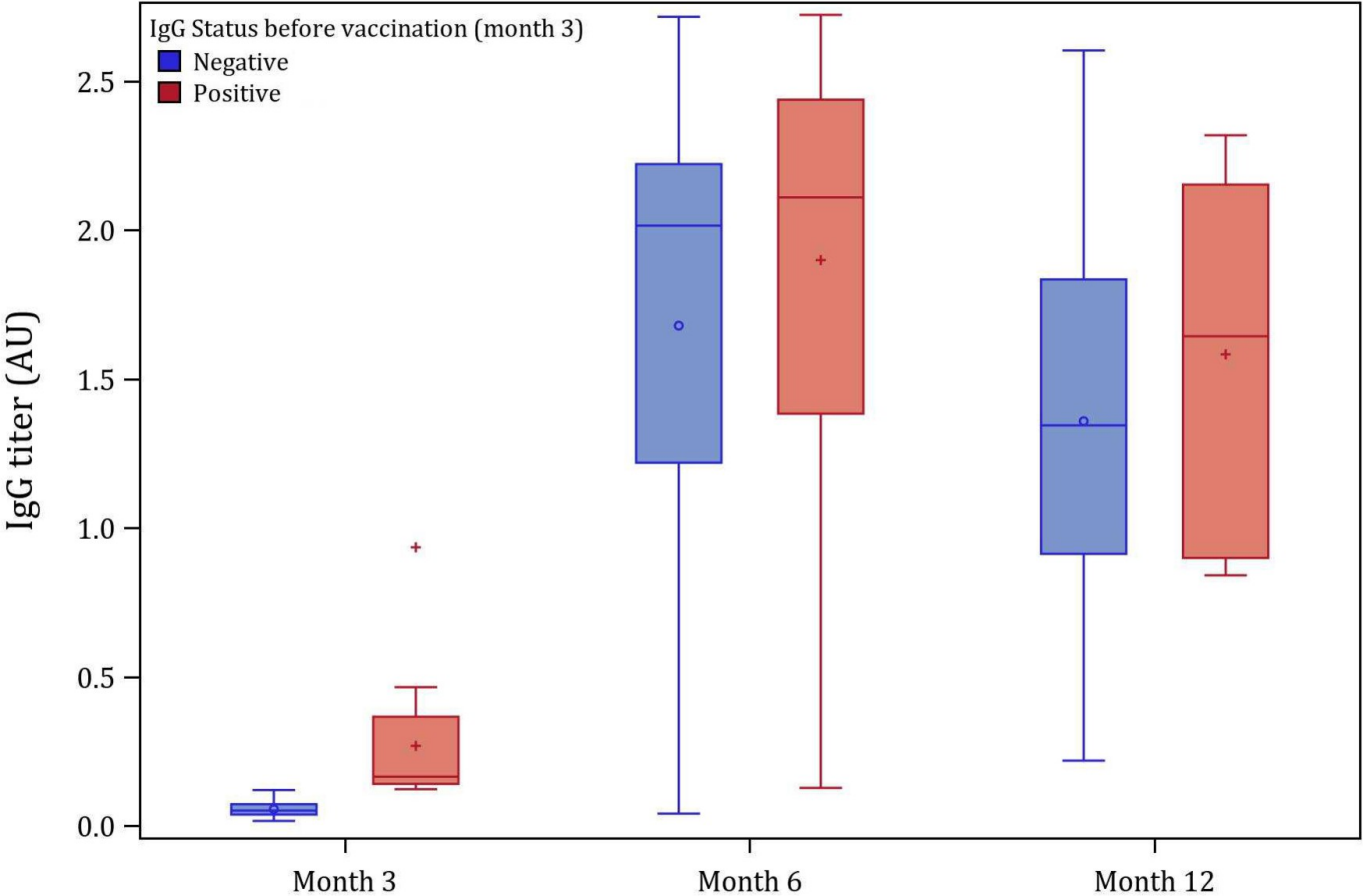
Box plot for participants who got blood tested at months 3, 6 and 12 comparing those spike protein IgG positive at month 3 versus those negative at month 3. Legend: The bottom and top edges of the box indicate the IQR, the marker inside the box indicates the mean value, the line inside the box indicates the median value. The upper and lower fences are maximum and minimum values, respectively, points beyond upper and lower fences are outliers. Red bar are those IgG positive at month 3 and blue bar are those negative at month 3.

The SARS-CoV-2 spike protein IgG titers from presumed exposure or infection with SARS-CoV-2 was markedly lower than from vaccination (Fig 3). Spike protein IgG titers for those vaccinated were generally 5.7 times higher than participants not vaccinated (median IgG titer = 0.37 (interquartile range [IQR] 0.17–1.1) arbitrary unit (AU) for positive antibody but not vaccinated versus 2.1 (IQR 1.5–2.3) for positive antibody and fully vaccinated) at 6 months. There was no significant difference (P = 0.20) in IgG levels after vaccination for patients who were spike protein IgG positive before vaccination (median IgG titer = 2.1 (IQR 1.4–2.4) versus those that were negative (median IgG titer = 2.0 (IQR 1.2–2.2) (Fig 4). However, level of IgG titers did wane over time after vaccination. At the 6 months blood draw, the median IgG titer for fully vaccinated participants was 2.1 (IQR 1.9–2.3). while at the 12 months blood draw, median IgG titer decreased to 1.33 (IQR 0.91–1.84).

### Factors associated with seropositivity prior to vaccination

Tables 2 and S1 include potential risk factors for either SARS-CoV-2 exposure and/or significant disease prior to availability of vaccines. During both the May to July 2020 (Table 2) and September to October 2020 (S1 Table) blood draws, there was no significant associations between age, gender, race, or presence of comorbidities and seropositive rates. During the September to October 2020 blood draw, those involved in direct patient care were significantly less like to be IgG seropositive (odds ratio (OR) 0.45, 95% CI 0.20–0.97, P = 0.04) and those working on site either 1–2 or 3 plus days per week were nonsignificantly less likely to be seropositive (OR 0.66, 95% CI 0.25–1.74 and 0.71, 95% CI 0.31–1.60 for 1–2 days and 3 or more days per week, respectively).

**Table 2:** Associations between presence of antibodies and selected factors at baseline (N = 579).

Factor		Positive IgG (N = 55)	Negative IgG (N = 524)	Odds Ratio (95% CI)	P value*
Age–years ^§^	Median (IQR)	35 (26–53)	40 (29–53)	0.99 (0.97, 1.0)	0.32^¶^
Sex	Female	47 (85%)	411 (79%)	1.60 (0.74, 3.49)	0.24
	Male	8 (15%)	112 (21%)	Ref	
Race	White	46 (84%)	445 (85%)	0.91 (0.43, 1.93)	0.80
	All other races	9 (16%)	79 (15%)	Ref	
Involved in direct patient care	Yes	17 (31%)	213 (41%)	0.65 (0.36, 1.19)	0.16
	No	38 (69%)	311 (59%)	Ref	
Number of days per week working on site	3 +	19 (36%)	239 (46%)	0.67 (0.35, 1.31)	0.37
	1–2	15 (28%)	121 (23%)	1.05 (0.51, 2.15)	
	0	19 (36%)	161 (31%)	Ref	
Comorbidities ^ǁ^	Yes	14 (25%)	107 (20%)	1.33 (0.7, 2.53)	0.38
	No	41 (75%)	417 (80%)	Ref	
Travel outside of the United States since Nov 2019	Yes	12 (22%)	147 (28%)	0.71 (0.37, 1.39)	0.32
	No	43 (78%)	376 (72%)	Ref	
Travel outside of Massachusetts since Jan 2020	Yes	40 (73%)	365 (70%)	1.16 (0.62, 2.16)	0.64
	No	15 (27%)	159 (30%)	Ref	

Participants who didn’t get vaccinated at time of blood drawl were included in the analysis.

Number of missing: Age = 5, Sex = 1, Number of days per week working on site = 5, Travel outside of the United States = 1.

^§^ Odds ratio per unit increase.

Odds ratios were estimated from logistic regression.

*P-value from the chi-square test unless specified.

^¶^ P-value from Wilcoxon rank sum test.

^ǁ^ Comorbidities including diabetes, hypertension, cardiovascular disease, asthma, chronic lung disease, chronic kidney disease, liver disease, cancer or autoimmune disease.

## Discussion

The first detection of COVID-19 infections in Massachusetts was in March 2020 leading to a spike in cases [8]. The capacities for PCR-based testing for active infection were insufficient to provide an accurate picture of the growing health crisis in the early weeks of the pandemic in the city [9]. This longitudinal study of employees in a comprehensive cancer center in Boston, MA was conducted during the first year of the COVID-19 pandemic and provides a retrospective analysis of the seroprevalence of SARS-CoV-2 amongst healthcare workers, insights into the effectiveness of protective measures rapidly implemented in March 2020, and the impact of the health care workplace safety measures compared to living in the local environment outside the Dana-Farber Cancer Institute. While an initial IgG seropositivity of 9.2% for the first blood draws between May and July 2020 indicates a substantial number of infected individuals compared to the expected by state-wide results from the limited PCR testing results, the rate of IgG seropositive to SARS-CoV-2 remained under 10% for the remaining year until the availability of vaccines. The relatively modest growth in seropositivity is likely due to a combination of factors including the public health measures taken by the state of Massachusetts, the COVID-19 counter-measures put in effect by Dana-Farber Cancer Institute (and other health care facilities) and the cohort biased by informed health care workers. Further, participants with direct patient contact were > 50% less likely to be seropositive prior to vaccination, potentially a further confirmation of the measure health care workers took to protect themselves and ultimately patients.

While limited in size, the study confirms the SARS-CoV-2 IgG response is persistent over time for convalescent individuals with seroconversion prior to vaccine availability. The fact that vaccine availability overlapped with study design and the quantitative nature of our serological assay, allowed us to compare the IgG response between convalescent and vaccinated individuals. We find that vaccinated individuals mount average titers 7.9 fold above the level observed with convalescent individuals shortly after vaccine administration. Importantly, despite the limitations of self-declaring vaccination status, the seropositivity of vaccinated individuals with 98% and 100% for individuals receiving a minimum of one dose or two doses, respectively, is in line with clinical trial data for the vaccines administered and suggests near complete induction of a humoral immune response for at least 6 months. Waning of levels of IgG seropositivity was detected over time, though the titers still remained higher than immunity from infection.

Other limitations of the study include that participation was voluntary and participation may be biased to individuals without symptomatic COVID and we are unable to linkage to employee records. However, Dana-Farber Cancer Institute employees were required to report positive COVID testing to occupational health. As of end of May 2021, 454 total employees (6.2%) reported positive COVID testing. The participants were predominantly female, relatively young in age, and had high vaccination early in the rollout of vaccine. While these factors may be considered limitations in generalizability, they are reflective of the demographics many health care facilities.

The present study shows that already early in 2020 a significant (~ 10%), largely undetected, seroprevalence of SARS-CoV-2 IgG existed amongst the Dana-Farber workforce. Importantly, the fraction of SARS-CoV-2 IgG seropositive individuals remained controlled up to the availability of SARS-CoV-2 vaccines in December 2020 suggesting that the measures taken to control infections as well as local testing strategies were adequate to protect the workforce and patients. The study also finds that mRNA vaccines lead to on average 7.9 fold higher SARS-CoV-2 IgG titers when compared to convalescent individuals.

## Supporting information

S1 Fig Sensitivity and specificity of ELISA IgG/IgM/IgA assay performed on a cohort of 68 SARS-CoV-2 PCR positive samples (CoV2+), and 100 pre-pandemic negative samples (healthy). Legend: Note: The ELISA was performed on singlicate sample, N = 1.
(TIF)

S1 Table Associations between presence of antibodies and selected factors at month 3, N = 429. (DOCX)

S1 File Anonymized data file to reproduce results. (XLSX)
