# Supplementary figures and images for: Serological testing for SARS-CoV-2 antibodies of employees shows low transmission working in a cancer center
Source: PLoS One. 2022 Apr 12;17(4):e0266791. doi: 10.1371/journal.pone.0266791 (PMC9004747; doi:10.1371/journal.pone.0266791)

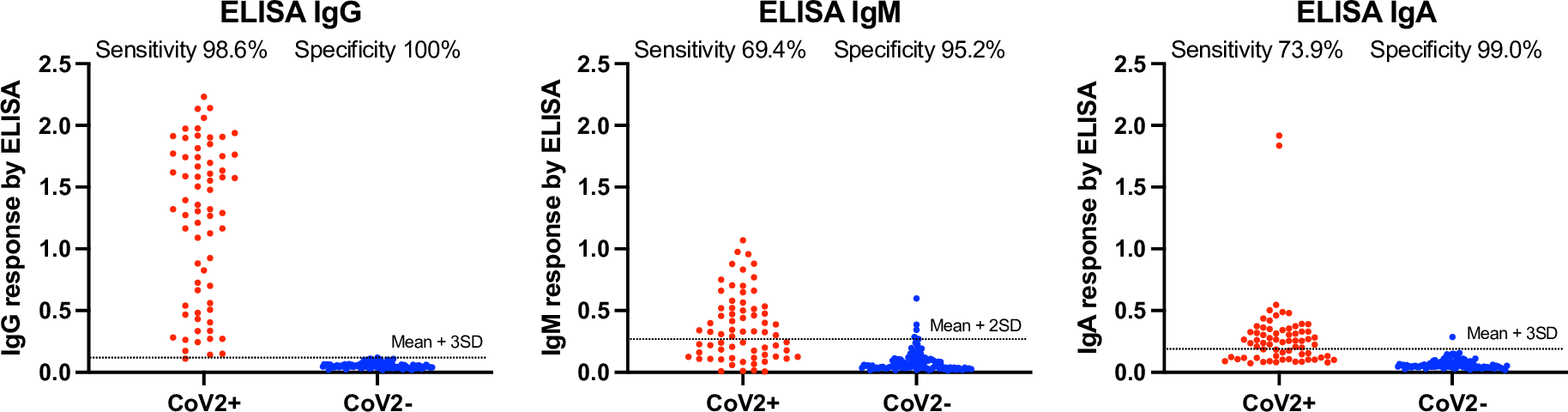

Supplement: S1 Fig — Legend: Note: The ELISA was performed on singlicate sample, N = 1. (TIF) [file pone.0266791.s001.tif]
