# Supplementary material for: Serological testing for SARS-CoV-2 antibodies of employees shows low transmission working in a cancer center
Source: PLoS One. 2022 Apr 12;17(4):e0266791. doi: 10.1371/journal.pone.0266791 (PMC9004747; doi:10.1371/journal.pone.0266791)
Supplement: S1 Table — (DOCX) [file pone.0266791.s002.docx]

| **Supplementary Table 1. Associations between presence of antibodies and selected factors at month 3, N = 429** | | | | | |
| --- | --- | --- | --- | --- | --- |
| Factor |  | Positive IgG  (N =36) | Negative IgG  (N =393) | Odds Ratio  (95% CI) | P value^*^ |
| Age – years ^§^ | Median (IQR) | 36 (29-52) | 40 (29-54) | 0.99 (0.97, 1.02) | 0.53^¶^ |
|  |  |  |  |  |  |
| Sex | Female | 32 (89%) | 309 (79%) | 2.15 (0.74, 6.25) | 0.16 |
|  | Male | 4 (11%) | 83 (21%) | Ref |  |
|  |  |  |  |  |  |
| Race | White | 29 (81%) | 342 (87%) | 0.62 (0.26, 1.48) | 0.28 |
|  | All other races | 7 (19%) | 51 (13%) | Ref |  |
|  |  |  |  |  |  |
| Involved in direct patient care | Yes | 9 (25%) | 168 (43%) | 0.45 (0.20, 0.97) | 0.04 |
|  | No | 27 (75%) | 225 (57%) | Ref |  |
|  |  |  |  |  |  |
| Number of days per week working on site | 3 + | 18 (50%) | 208 (53%) | 0.71 (0.31, 1.60) | 0.63 |
|  | 1-2 | 8 (22%) | 100 (26%) | 0.66 (0.25, 1.74) |  |
|  | 0 | 10 (28%) | 82 (21%) | Ref |  |
|  |  |  |  |  |  |
| Comorbidities ^ǁ^ | Yes | 9 (25%) | 84 (21%) | 1.23 (0.56, 2.71) | 0.61 |
|  | No | 27 (75%) | 309 (79%) | Ref |  |
|  |  |  |  |  |  |
| Travel outside of Massachusetts in past 3 months | Yes | 23 (64%) | 269 (68%) | 0.82 (0.4, 1.66) | 0.57 |
|  | No | 13 (36%) | 124 (32%) | Ref |  |

Participants who didn’t get vaccinated at time of blood drawl were included in the analysis.

Number of missing: Age = 5, Sex = 1, Number of days per week working on site = 3.

^§^ Odds ratio per unit increase.

Odds ratios were estimated from logistic regression.

^*^P-value from the chi-square test unless specified.

^¶^ P-value from Wilcoxon rank sum test.

^ǁ^ Comorbidities including diabetes, hypertension, cardiovascular disease, asthma, chronic lung disease, chronic kidney disease, liver disease, cancer or autoimmune disease.
